# Probing the Reactivity of ZnO with Perovskite Precursors

**DOI:** 10.1021/acsami.4c01945

**Published:** 2024-03-14

**Authors:** Sofia Apergi, Geert Brocks, Shuxia Tao, Selina Olthof

**Affiliations:** † Materials Simulation and Modelling, Department of Applied Physics, Eindhoven University of Technology, P.O. Box 513, 5600 MB Eindhoven, The Netherlands; ‡ Computational Chemical Physics, Faculty of Science and Technology and MESA+ Institute for Nanotechnology, University of Twente, P.O. Box 217, 7500 AE Enschede, The Netherlands; § University of Cologne, Institute for Physical Chemistry, Greinstrasse 4-6, 50939 Cologne, Germany; ∥ Center for Computational Energy Research, Department of Applied Physics, Eindhoven University of Technology, P.O. Box 513, 5600 MB Eindhoven, The Netherlands

**Keywords:** metal oxide, XPS, ZnO, perovskite, DFT, reactivity, interface

## Abstract

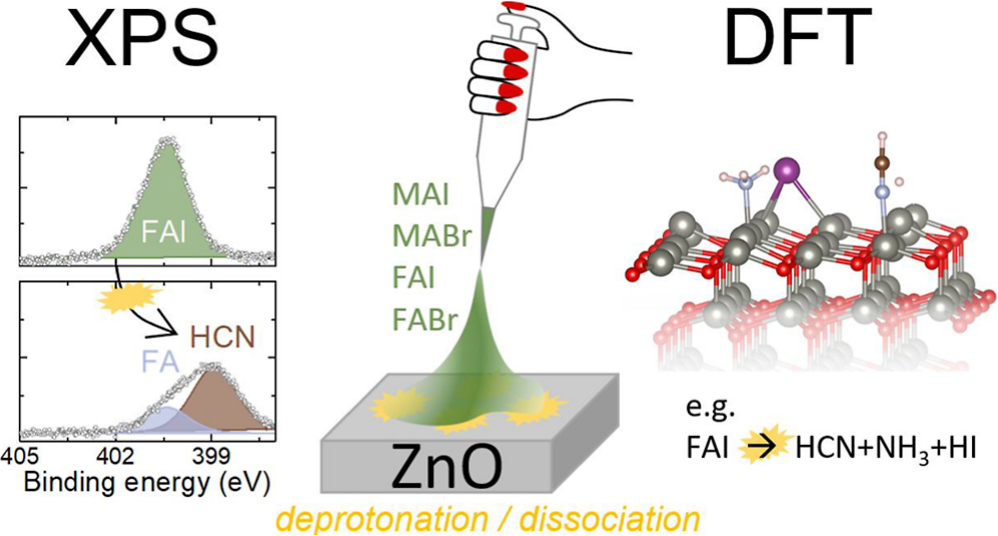

To achieve more stable and efficient metal halide perovskite devices, optimization of charge transport materials and their interfaces with perovskites is crucial. ZnO on paper would make an ideal electron transport layer in perovskite devices. This metal oxide has a large bandgap, making it transparent to visible light; it can be easily n-type doped, has a decent electron mobility, and is thought to be chemically relatively inert. However, in combination with perovskites, ZnO has turned out to be a source of instability, rapidly degrading the performance of devices. In this work, we provide a comprehensive experimental and computational study of the interaction between the most common organic perovskite precursors and the surface of ZnO, with the aim of understanding the observed instability. Using X-ray photoelectron spectroscopy, we find a complete degradation of the precursors in contact with ZnO and the formation of volatile species as well as new surface bonds. Our computational work reveals that different pristine and defected surface terminations of ZnO facilitate the decomposition of the perovskite precursor molecules, mainly through deprotonation, making the deposition of the latter on those surfaces impossible without the use of passivation.

## Introduction

The recent emergence of metal halide perovskites as potential photovoltaic absorbers^1^ and the intense research effort that followed^2^ has led to a remarkable improvement of their already impressive optoelectronic properties, while the range of possible applications keeps expanding and now includes light emitting diodes,^3^ photocatalysts^4,5^ and memristors,^6^ among others. Despite the progress, perovskites still suffer from severe instability that prohibits their commercialization.^7−9^ To this end, significant progress has been made on the bulk of the material, by means of compositional^10^ and dimensional^11^ engineering, as well as by optimization of fabrication techniques,^10,12^ but with regards to the instability at interfaces in perovskite devices, there is plenty of room for improvement.

In a typical perovskite device, the perovskite active layer is surrounded by a series of charge selective contacts, whose main purpose is to drive the charge carriers in the desired direction. In a solar cell, for instance, the charge carriers need to be extracted from the perovskite layer, while in a light emitting diode they have to be injected.^13,14^ An ideal charge transport layer must combine sufficient electrical conductivity, suitable energy levels, and good stability under illumination and thermal stress.

Here, metal oxides are an attractive option.^15^ They typically have large band gaps, making them transparent to visible light, which is necessary for applications in photovoltaics and light-emitting diodes. Depending on their native defects, such as metal or oxygen vacancies, some of these metal oxides end up inherently p-doped or n-doped, which makes them potential candidate hole and electron transport layer (HTL and ETL) materials, respectively. Indeed, many have been utilized successfully, not only in the field of halide perovskites, but also in organic optoelectronic devices^16^ and dye-sensitized solar cells.^17^ One of the most widespread ETLs by far is TiO_2_, but its rather low electron mobility leads to charge accumulation at the interface with the absorber layer^18^ when employed in halide perovskite solar cells. ZnO has become more popular in recent years in the field of organic optoelectronics, due to its superior electronic properties,^19,20^ but it unfortunately leads to a rapid decomposition of adjacent perovskite layers.^21−23^ Such a degradation not only occurs with ZnO; the use of metal oxides in contact with perovskites has turned out to be problematic more generally. For example, Hsu et al. showed the degradation of the organic perovskite precursors MAI (CH_3_NH_3_I) and FAI (CHN_2_H_4_I) in contact with NiO, TiO_2_, and SnO_2_ using infrared spectroscopy and mass spectrometry as well as density functional theory (DFT) calculations.^24,25^ Surface-sensitive methods like X-ray photoelectron spectroscopy (XPS) are also commonly used to study degradation at perovskite interfaces with metal oxides, such as NiO,^26^ TiO_2_,^27^ ITO,^28^ and MoO_3_.^28−31^ The majority of the degradation processes were found to be connected with the decomposition of the organic precursors. In some cases, such as MoO_3_, the metal oxide also undergoes redox reactions with the halide species.^29^

In this work, we provide a comprehensive study of the interaction between the most common methylammonium (MA) and formamidinium (FA)-based organic perovskite precursors, MAI, FAI, MABr, and FABr with ZnO, by combining the analysis of the interfaces by XPS with DFT calculations. For the DFT calculations, we investigate several different possible ZnO surface terminations, both polar and nonpolar. In addition, we examine the role of oxygen vacancies as well as hydroxylation or water absorption on the ZnO surface. By making this comprehensive comparison, we can confirm that all ZnO terminations seem to be equally reactive. Therefore, ZnO consistently facilitates the decomposition of the organic perovskite precursors, which mainly takes place via deprotonation but also through more complex dissociation reactions. The reactivity scales to some degree with the choice of precursor, MAI being the most reactive, followed by MABr, FAI, and finally FABr.

## Results and Discussion

### Experimental Results

**Figure 1:**
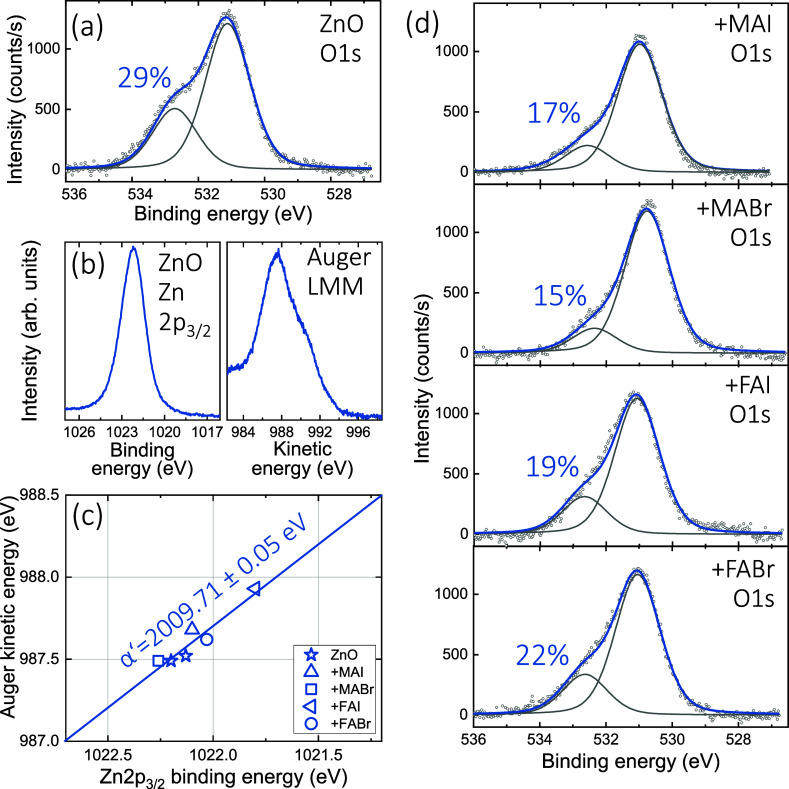
XPS data related to the ZnO surface. (a) Oxygen 1s signal of an as-prepared sample with the amount of OH-related signal indicated in the graph. (b) Zn-related 2p_3/2_ core-level peak and Auger LMM signal. (c) Wagner plot of the as-prepared ZnO as well as the ZnO samples treated with the different precursors, showing that all data points fall on a common line and therefore have the same Auger parameter α′, as indicated in the graph. (d) O 1s signal for the ZnO surfaces treated with the different precursors.

First, we will present XPS measurements of solution-processed ZnO surfaces before and after exposure to the precursors MAI, MABr, FAI, or FABr; these results will be later compared to the insights gained by DFT calculations. Details of the sample preparation and experimental technique can be found at the end of the document. Figure 1a shows the oxygen 1s core-level signal of the as-prepared ZnO surface, which is fitted with two distinct contributions. The main signal at lower binding energy (*E*_B_) on the right corresponds to oxygen incorporated in the ZnO lattice, while the one at higher binding energy is commonly associated with surface OH species.^32^ These OH groups can have their origin in the air exposure of the film or could be a remnant from the preparation procedure if the used zinc acetate dihydrate is not completely transformed. Measurements of various similarly prepared ZnO samples show a concentration of O groups in the range of 25–30% using this surface-sensitive XPS technique. The Zn 2p_3/2_ core-level peak can be found in Figure 1b, together with the Zn Auger L_3_M_4,5_M_4,5_ signal. For measurements of ZnO, it is important to report both of these signals together, since the identification of the oxidation state or changes thereof is not possible using the 2p core level signal alone.^33^ Auger transitions are more sensitive to the chemical environment, leading to a more pronounced shift of the LMM Auger peak compared to the 2p core level.^34^ The so-called Auger parameter α′ can be extracted, which is the sum of the kinetic energy (*E*_K_) of the Auger peak and the binding energy of the core level peak, i.e., α′ = *E*_K_ (Auger)+ *E*_B_ (core level). For the data set shown here, we find *E*_B_(Zn 2p_3/2_) = 1022.19 eV, *E*_K_(Zn LMM) = 987.49 eV and therefore α′ = 2009.68 eV. This is a slightly lower value than, for example, reported by Biesinger et al. for ZnO (α′ = 2010.4 eV),^33^ but the reported are spread over quite a range.^35^ As we use this parameter here to identify changes in the chemical state of Zn due to the interaction with the perovskite precursors, only changes in the value of α′ are relevant here.

We exposed such ZnO samples to a small amount of one of the four different perovskite precursors (MAI, MABr, FAI, or FABr), dissolved at a low concentration of 0.05 molar in isopropanol alcohol, by spin-coating them onto the ZnO surface. The XPS measurements of the Zn related features show slight variations in binding energy, likely due to changes in the work function or doping level; the data are included in the Supporting Information Figure S1. The extracted core and Auger level positions are summarized in Figure 1c, in a so-called Wagner plot that shows the Zn 2p_3/2_ binding energy on the abscissa and the Auger kinetic energy on the ordinate. All data points fall on a line with an average value of α′ = 2009.71 ± 0.05 eV; therefore, no significant changes are found for the Zn-oxidation state. This is in clear contrast to our previous study on MoO_3_, where we found Mo to be reduced due to redox reactions taking place on the sample surface.^29^

The oxygen signals after the precursor treatment are shown in Figure 1d. A clear reduction in the amount of surface OH groups is observed as a consequence of the interaction with the precursors, indicating that some of these OH groups are removed. The underlying mechanism will be made clear in the discussion below.

**Figure 2:**
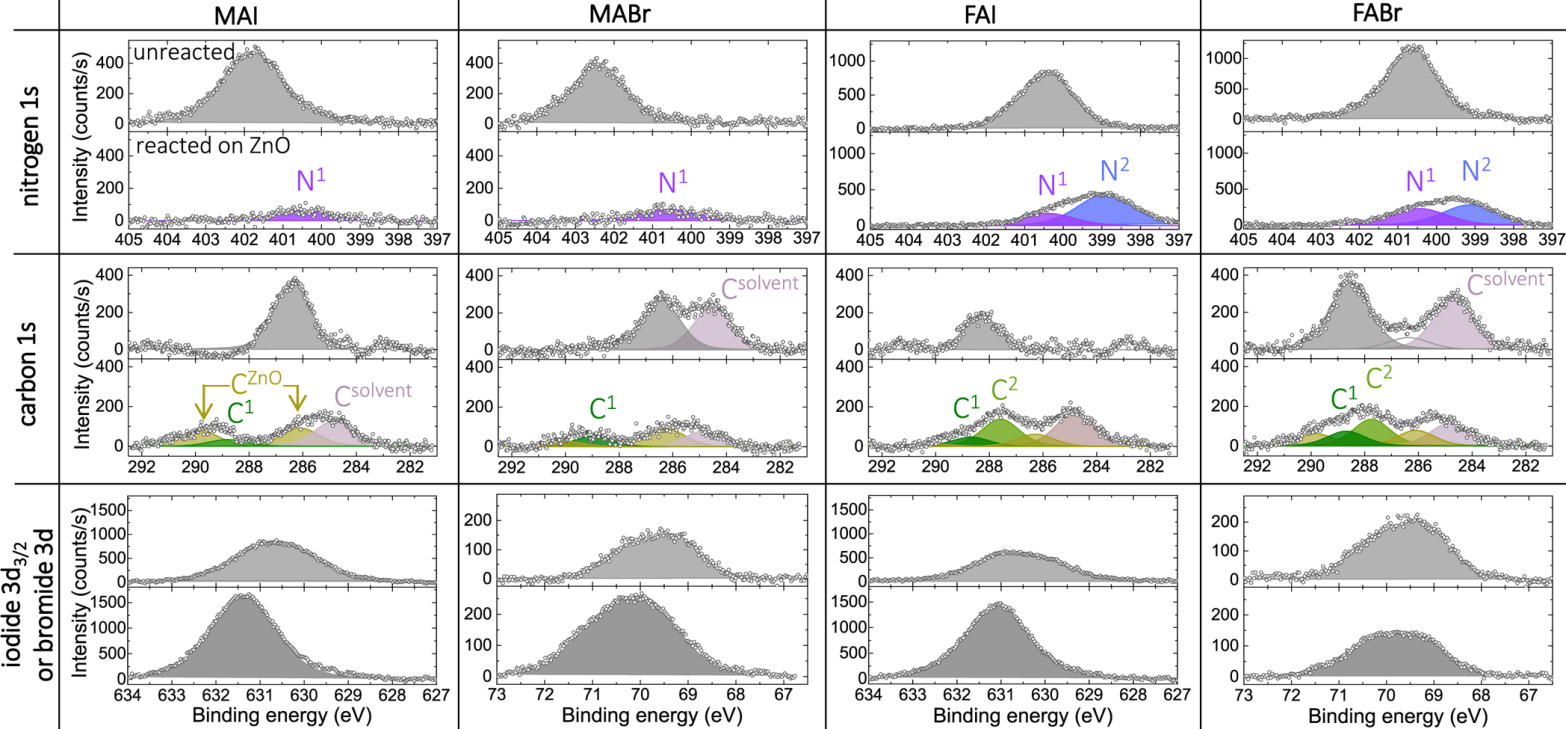
XPS core-level signals of elements related to the precursors. The columns show the data belonging to the different precursors MAI, MABr, FAI, and FABr, while the lines correspond to the N 1s, C 1s, as well as I 3d_3/2_/Br 3d signals. In each of the graphs, the upper panel represents the unreacted, i.e., intact, precursor signal, while the lower one shows the degraded precursor in contact with ZnO.

As we are interested in the degradation reactions of the precursors, we also probed the core level peaks of carbon, nitrogen, and the halides; the data are summarized in Figure 2. Here, the columns correspond to the different precursors, while the rows present the elements. In the following, we will only discuss the observations made with XPS, while the discussion of the underlying mechanisms leading to the formation of the degraded or volatile species will follow below, based on the insights gained by the DFT analysis.

To identify the formation of new surface bonds or the creation of volatile (and therefore missing) species, reference data sets of unreacted precursor films are needed at the same molar concentration. No common inert substrate could be found for all four precursors; therefore, MAI and FAI were deposited on PEDOT/PSS, while MABr and FABr were deposited on ZnCl_2_. The integrity of the precursors was confirmed by determining the elemental composition of the films, which were close to the expected values. These reference data sets are shown in the upper panel of each of the subfigures in Figure 2; the lower panels show the deposition of the same precursor solutions onto ZnO, where significant degradation occurs.

The top row of Figure 2 shows the nitrogen 1s signal. Here, the *y*-scale for FA containing precursors is approximately twice that for MA, as expected due to the different molecular structure. Deposited onto ZnO, all N signals significantly decrease, which is indicative of the formation of volatile species that are leaving the surface. In the case of MAX, around 75% of the N is lost, while this loss is lower for FAX, namely, 30% in the case of FAI and 55% for FABr. In all cases, the binding energy of the remaining N signal changes significantly. For the unreacted MAI and MABr layers, the N 1s peaks are located at 401.8 and 402.4 eV, respectively, while upon deposition on ZnO, both show a common value of around 400.5 eV, labeled as N^1^ in Figure 2. The same feature is observed for the FA-containing precursors, indicating that the same degraded species is created and adsorbs on ZnO for all four precursors. In addition, a second feature is observed for the FA-containing precursors, labeled N^2^, at an even lower binding energy of ∼399 eV, which suggests that here an additional degraded species sticks to the surface.

The carbon signal is considered next. It has to be noted that the C 1s data for the unreacted MAI and FAI samples have been modified by subtracting the signal originating from the underlying PEDO/PSS layer, as otherwise the subtle precursor signal would not have been discernible in the plot. The original data and fitting procedure are included in the Supporting Information and Figure S2. For the Br-containing precursor species deposited on ZnCl_2_, the subtraction of a substrate signal was not necessary, but a contamination- or solvent-related additional C peak at 284.5 eV is observed, marked as C^solvent^. For the degraded precursors in contact with ZnO, we see several distinct carbon related peaks. One of them is again solvent-related, while the features marked by C^ZnO^ originate from the underlying ZnO substrate. A more detailed discussion on the origin of these C^ZnO^ features can be found in the Supporting Information, Figures S3 and S4. These C signals from the ZnO surface remain visible since the precursor coverage is lower than the probing depth of the XPS measurement. In addition, two new carbon bonds are formed. The one labeled C^1^ is located around 288.8 eV and is present for all precursors, while a second bond labeled C^2^ is seen only for FA-containing precursors at ∼287.6 eV. Not considering the ZnO or solvent-related C signals, we also see a significant loss of carbon species in contact to ZnO for most precursors. While for FAI, most of the carbon seems to remain on the surface in one of the two new bonds, the loss is 90, 80, and 50% for MAI, MABr, and FABr, respectively.

Finally, for the halides, the iodide 3d_3/2_ and the bromide 3d doublet are shown in the bottom row, where we do not observe a loss in signal intensity. On the contrary, most precursors show a significantly higher halide signal in contact with ZnO, which is an indication of the formation of surface bonds. The binding energies remain similar to those of the unreacted precursors, indicating a similar oxidation state of I^–^ and Br^–^.

From the experimental data shown in Figure 2, it is evident that the ZnO surface is highly reactive, leading to the formation of degradation products that either absorb on the surface or leave as volatile compounds. In order to identify the reaction pathways and degradation products, we conducted a comprehensive computational study that is discussed next.

### Computational Results

To analyze the reactivity between ZnO and the precursors MAI, FAI, MABr, and FABr, we examine how easily the precursor molecules decompose on the surface of the oxide. Following previous studies,^25,29,36,37^ we choose two possible decomposition reaction paths for MAX and two for FAX. The corresponding reaction energies on different surface terminations of ZnO were calculated and compared to the reaction energies for the free-standing molecules. The four studied reactions are
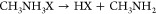
A1and
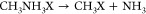
A2for MAX and
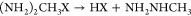
B1and
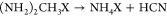
B2for FAX. Reactions A1 and B1 are simple deprotonation reactions, where the cations MA^+^ and FA^+^ lose a proton to the surface of ZnO and form the neutral molecules MA and FA respectively. Reactions A2 and B2 are more complex dissociation reactions involving breaking of C–N bonds.

#### Pristine ZnO Surface

**Figure 3:**
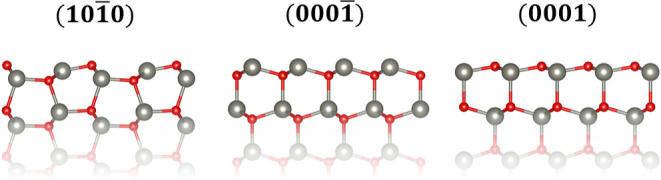
Atomistic representation of the studied ZnO terminations. The Zn atoms are represented by the gray smaller balls and the oxygen atoms by the red balls.

We begin our study with the clean nonpolar (101̅0) as well as the polar Zn-terminated (0001̅) and O-terminated (0001) surfaces, as shown in Figure 3. The former is an obvious choice, since it is thermodynamically the most stable ZnO surface termination.^32^ As for the polar ZnO surfaces, their stability has been a long-standing issue, and no consensus has been reached yet concerning their microscopic structure. Most likely, the stability of polar ZnO surfaces stems from surface reconstructions and/or from the adsorption of charged species like H^+^ or OH^–^.^32^ Many of the proposed reconstructions^38−40^ are quite complex, and their study requires the use of large, computationally expensive, supercells, which would prohibit the study of reactions on such surfaces. More recently, Mora-Fonz et al.^41^ suggested the existence of multiple possible microscopic configurations of similar energy for both Zn-terminated (0001̅) and O-terminated (0001) polar surfaces, some of which are not as severely reconstructed as previously thought. In the present study, we are mainly interested in comparing the reactivity of the polar surfaces to that of the nonpolar (101̅0) ZnO surface, so we choose to limit ourselves to the clean unreconstructed (0001̅) and (0001) surfaces. In addition, surfaces with defects or hydroxyl and water adsorbents are discussed in the next section.

In Table 1, the calculated reaction energies for reactions A1–B2 are presented for the free-standing molecules, as well as for the same molecules on the (101̅0), (0001̅), and (0001) surfaces of ZnO. These reaction energies are calculated as *E*_reac_ = *E*_fi_ – *E*_init_, where *E*_fin_ is the total energy of the reaction product molecules and *E*_init_ is the energy of the reactants. For the free-standing molecules, all reaction energies are positive, ranging from 0.24 to 1.55 eV, indicating that these molecules are stable. Indeed, experimentally, their decomposition is only observed at elevated temperatures.^25^ Both deprotonation and dissociation are more likely for MAX than for FAX. The difference in ease of deprotonation correlates with the p*K*_a_’s of MA and FA. In water, the values are p*K*_a_ = 10.66 for MA^42^ and 12.52 for FA,^43^ so the protonated FA is somewhat more stable than the protonated MA. As for the dissociation, the double C–N bond in FAX is much harder to break than the single bond in MAX, making the dissociation of FAX the least probable of these studied reactions.

**Table 1:** Reaction Energies in eV of the Deprotonation and Dissociation Reactions of Free-Standing AX Molecules and for Molecules and Reaction Products Adsorbed on the Clean ZnO (101̅0), (0001̅), and (0001) Surfaces

	free-standing		ZnO (101̅0)		ZnO (0001̅)		ZnO(0001)	
	A1/B1	A2/B2	A1/B1	A2/B2	A1/B1	A2/B2	A1/B1	A2/B2
**MAI**	0.65	0.24	–1.46	–0.68	–1.69	–2.15	–1.33	–0.63
**FAI**	1.08	1.55	–1.04	–0.28	–1.13	–0.31	–1.15	0.25
**MABr**	0.64	0.34	–1.35	–0.57	–1.61	–2.09	–1.31	–0.64
**FABr**	1.05	1.48	–0.98	–0.20	–1.09	–0.25	–1.14	0.27

On the surface of ZnO, the calculated values change drastically. On (101̅0) ZnO, all reaction energies are negative, ranging from −1.46 to −0.20 eV, meaning that the surface promotes these reactions. Notably, when MAX is put on top of the surface and the structure is left to relax, deprotonation of the organic cation is spontaneous. In order to be able to calculate reaction energies for establishing a trend, we therefore “freeze” the –NH_3_ tail of MAX and repeat the calculation (Figure S5 in the Supporting Information). The calculated deprotonation energy of MAI (reaction A1) is the lowest of the reaction energies on (101̅0) ZnO, with the deprotonation energy of MABr being only slightly higher. The deprotonation of FAI and FABr (reaction B1) follows, also with very low energies around −1 eV. It should be noted that in all cases, the proton and the halide are adsorbed separately on the surface. The proton adsorbs on a surface oxygen, turning it into an OH group, and the halide adsorbs on a neighboring surface Zn atom.

**Figure 4:**
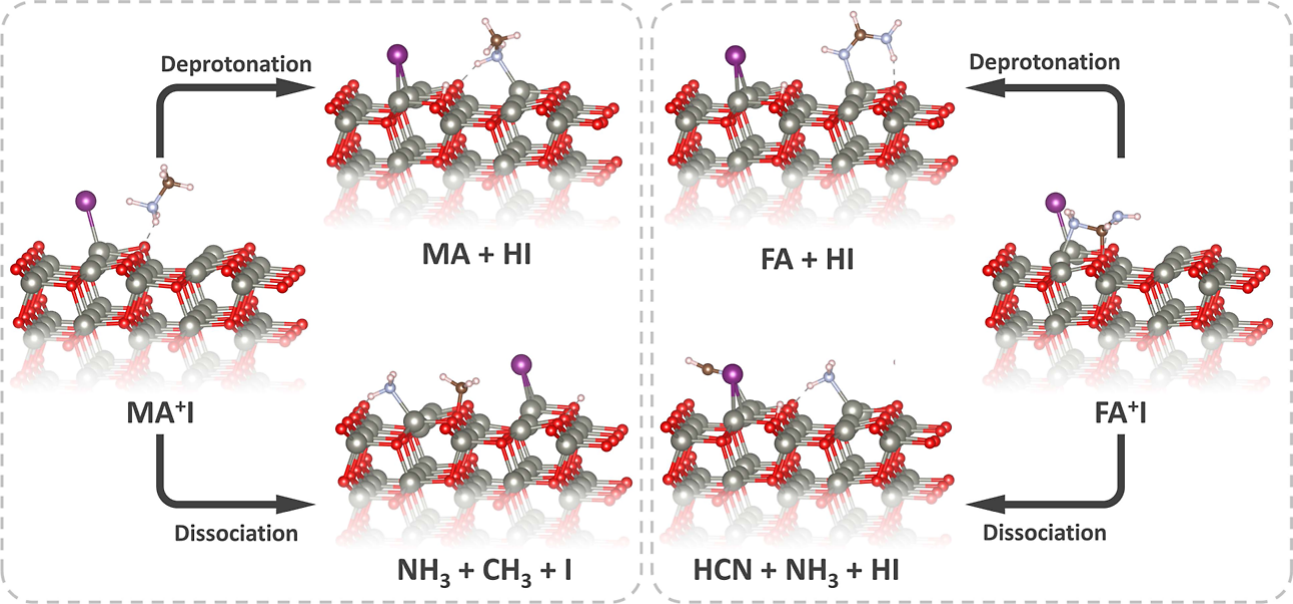
Atomistic representation of reactions A1–B2 on the surface of pristine nonpolar (101̅0) ZnO.

The dissociation energies of all four molecules (reactions A2 and B2) are much higher than those of the deprotonation (reactions A1 and B1), but they are still negative and lower than the corresponding energies for the free-standing molecules, meaning that these reactions are also likely to occur. It should be noted that for reaction A2 of MAX on (101̅0) ZnO, the reaction product CH_3_X further decomposes to CH_3_ and X, which are then adsorbed separately on the surface of the oxide. Without taking this into account, the calculated reaction energies for the dissociation of MAI and MABr on (101̅0) would be 0.60 and 0.72 eV respectively. Similarly, the product NH_4_X of reaction B2 decomposes to NH_3_ and H + X on ZnO. The atomistic representations of all reactions on the nonpolar surface of ZnO are shown in Figure 4.

The results on the polar (0001̅) surface are quite similar to the nonpolar (101̅0) surface. The overall trend is the same, with the exception of the dissociation of MAX (A2) now being more likely than the deprotonation (A1), which is the same as that for the free-standing molecules. To understand why this happens, we can look at the adsorption energies of the reaction products in Table S1 in the Supporting Information. On the nonpolar (101̅0) ZnO surface, HX (*E*_ads_ ≈ −2 eV, with the proton and the iodide ion again separating and finding favorable adsorption positions on surface O and Zn atoms, respectively) adsorbs more strongly than CH_3_X (*E*_ads_ ≈ −0.94 eV), while the adsorption energies of MA and NH_3_ are very similar (−1.25 and −1.19 eV respectively). This leads to the deprotonation being more favorable here since the corresponding reaction products have a stronger interaction with that surface.

In contrast, on the polar (0001̅) surface, the calculated adsorption energies of the products of both deprotonation and dissociation reactions are almost the same ( eV), in which case the trend that is observed for the gas phase prevails. Moreover, unlike the nonpolar surface, on the polar surface the deprotonation of MAX is not spontaneous. We correlate this with the fact that a polar surface offers favorable adsorption sites for only one type of ion, i.e., either cations or anions, but not for the other type, and, for instance, HX splits up into a proton and an iodide ion. In contrast, the nonpolar surface offers adsorption sites for both types of ions. The products of reactions A2 and B2 decompose further, same as for the (101̅0) termination. The atomistic representations of all reactions on the polar (0001̅) surface of ZnO are shown in Figure S6.

As for the O-terminated (0001) surface, the trend is again the same as for the nonpolar surface, with similar deprotonation energies for all compounds. The dissociation energies are also similar for both MAX molecules, while being less favorable for FAX on this surface, with positive reaction energies of around 0.26 eV. This discrepancy most likely stems from the lack of available Zn atoms on the surface with which the resulting HCN can bond, as discussed above. The result is a much weaker interaction of the molecule with this surface of ZnO, with an adsorption energy of −0.12 eV, as compared to −0.59 and −0.34 eV on the (101̅0) and (0001̅) surface, respectively. Still, the dissociation energy of FAX is more than 1 eV lower on this surface termination of ZnO, compared with the gas phase. The atomistic representations of all reactions on the polar (0001) surface of ZnO are shown in Figure S7 in the Supporting Information.

On all three surfaces, the order of reactivity for the four molecules is MAI ≈ MABr > FAI ≈ FABr. This can be explained based on p*K*_a_s, the same as for the free-standing molecules: MAX deprotonates more easily than FAX on the basic ZnO surface due to its lower p*K*_a_. As for the differences between I and Br, these are quite small and no significant trend is observed. We therefore conclude that the two halides behave quite similarly on the surface of ZnO. From the adsorption energies of the different molecules in Table S1 in the Supporting Information, we can see that some of the decomposition products adsorb very strongly on ZnO surfaces, most notably HX, but also NH_4_X and CH_3_X. This strong interaction of the reaction products with the oxide surface is another reason why the reaction energies are so low.

Based on the above, we can conclude that the pristine surface of ZnO is indeed not a good candidate for the deposition of organic halide perovskites since the decomposition of their organic precursors on that surface is certain. Next, we will explore the effect that some of the most commonly found defects on the ZnO surface have on the reactivity of the oxide. Since all three of the studied surfaces are equally reactive and exhibit similar trends, with the exception of the (0001̅) surface, on which the dissociation of MAX is more likely than the deprotonation, in the following, we will only focus on the nonpolar (101̅0) surface. We will investigate the role of oxygen vacancies as well as surface hydroxylation and water adsorption.

#### Defective ZnO Surface

We first consider the most common intrinsic defect found in ZnO, namely, O vacancies.^44,45^ In our previous study on the interaction between MoO_3_ and perovskite precursors, we found a significant lowering of all reaction energies due to the presence of O vacancies.^29^ This motivated the study of oxygen vacancies here, which we model by removing one O atom from the surface of (101̅0) ZnO.

**Figure 5:**
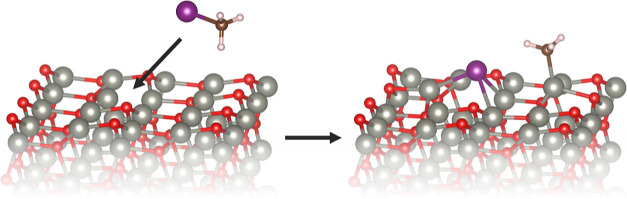
Adsorption geometry of CH_3_X on the (101̅0)-terminated ZnO surface with an O vacancy.

From the calculated reaction energies in Table 2, we can see that for ZnO, the presence of oxygen vacancies does not have a significant effect on the reactivity of the surface. The energies of reactions A1, B1, and B2 are almost the same as those in the pristine case. For reaction A2 however, slight differences are found. Specifically, the dissociation of MAX is now comparable in energy with the deprotonation, whereas in the pristine case, the deprotonation energy was much more negative than the dissociation. As can be seen in Figure S8, almost none of the reactant molecules or the degradation products interact with the O vacancy on the surface of ZnO. Instead, they are adsorbed preferably as far away as possible from the O vacancy. The only exception is CH_3_X, whose adsorption is more favorable at the vacancy. As before, CH_3_X splits up into CH_3_ and X, with the latter occupying the O vacancy site, and the former adsorbed on a Zn surface atom as shown in Figure 5. For this reason, the dissociation of MAX becomes more likely on the defective case than on the pristine case, while all other reactions are not affected by the presence of the vacancy.

**Table 2:** Reaction Energies in eV of the Deprotonation and Dissociation Reactions of Perovskite Precursor Molecules on ZnO (101̅0) with an O Vacancy

	A1/B1	A2/B2
**MAI**	–0.97	–0.94
**FAI**	–1.04	–0.32
**MABr**	–1.00	–0.89
**FABr**	–0.98	–0.26

#### Hydroxylation and Water Adsorption

Next, we investigate the reactivity of the hydroxylated (101̅0) surface. Modeling the process of synthesizing ZnO is not feasible from first principles. To have some idea of how hydroxyl groups at the surface might form, we use simple estimates based on ZnO in a water environment under equilibrium conditions. Pure hydroxylation is then unlikely, i.e., a ZnO surface fully or partially covered with OH groups only.^46^ This is because coverage by the OH groups requires a redox reaction that constitutes the first step in the oxygen evolution reaction (OER) of water splitting. The reaction involves the dissociation of water, leading to the generation of OH^–^, as well as the transfer of an electron to form OH. This does not happen spontaneously in the absence of an external potential. The absolute potential −4.44 eV at pH = 0 of the standard hydrogen electrode,^47^ minus the standard free energy of water splitting 1.23 eV, minus a calculated overpotential for this reaction step of ∼0.5 eV,^46^ puts the potential of this first OER step at −6.17 eV. Under strong alkaline conditions pH = 14, this potential moves up by 0.82 eV to *W*_eq_ = −5.35 eV. As the reported work function of ZnO is −4.95 ≤ *W*_F_ ≤ −3.50,^48,49^ it means *W*_F_ < *W*_eq_, so the oxidation of water molecules, required for OH adsorption, will not happen spontaneously.

On the other hand, adsorption of water molecules on the ZnO surface is very likely. We calculate the adsorption energy of a water molecule from the gas phase onto the ZnO surface as −0.96 ± 0.01 eV, with only a weak coverage dependence, indicating that water molecules bind strongly to the surface. With respect to water gas at pressure *p* = 0.035 bar, which is the equilibrium pressure of liquid water at standard conditions, the change in the entropy term TS is 0.67 eV/water molecule.^46^ The free energy change associated with the adsorption of water is then −0.29 eV/molecule, making adsorption very likely.

**Figure 6:**
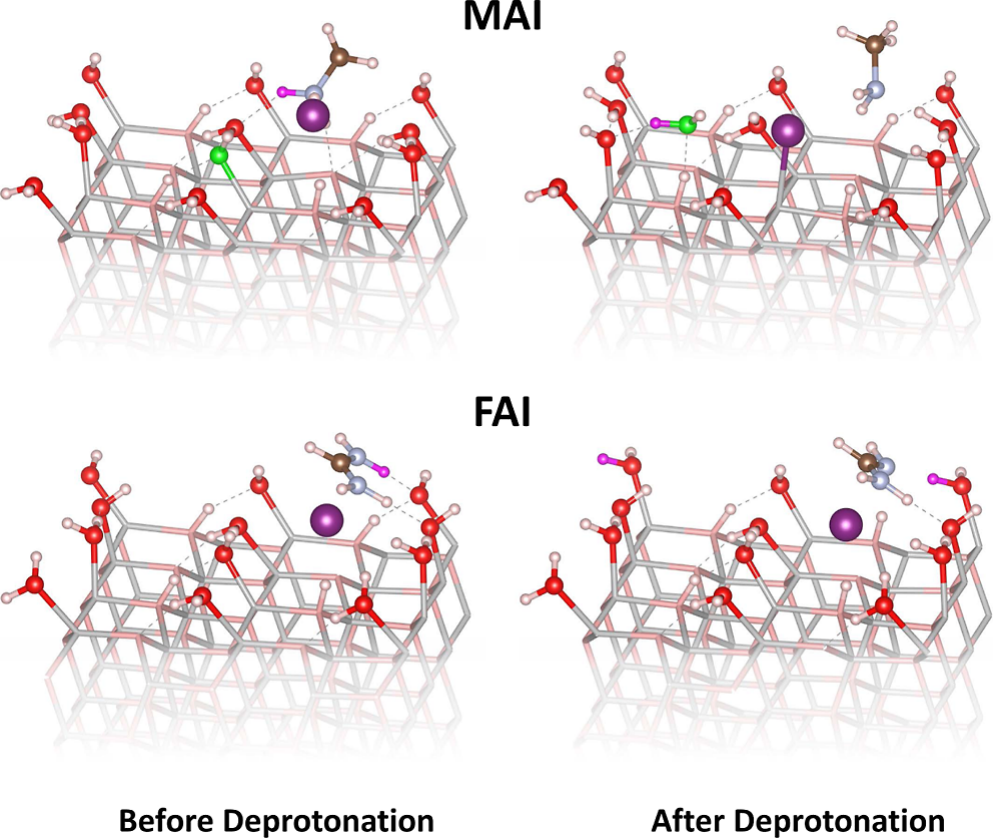
Atomistic representation of reactions A1 and B1 on the surface of hydroxylated nonpolar (101̅0) ZnO. The substrate has been faded for clarity.

If the coverage reaches a full monolayer, the system is additionally stabilized by 0.21 eV/molecule through dissociation of every other adsorbed water molecule into OH + H, where the split-off hydrogen atom adsorbs on a nearby surface oxygen atom, as shown in Figure 6; this is in agreement with the results of previous studies.^50,51^ In fact, this stabilization energy is sufficiently large to prevent homogeneous partial water coverage. Instead, the latter will phase separate into densely packed and empty surface patches. The former will then have the structure, as shown in Figure 6, with every second water molecule split into OH and H. This densely packed structure does not anymore provide enough adsorption sites for most of the studied molecules, thus inhibiting their interaction with the surface.

As we previously saw, the strong interaction of the reaction products with the surface is what drives the degradation of the perovskite precursors on ZnO; therefore, we do not expect this surface structure to be as favorable for the degradation reactions as the pristine surface. Indeed, on the water-covered surface, the deprotonation energies of MAI, FAI, MABr, and FABr are −0.09, 0.15, −0.08, and 0.15 eV respectively. As expected, the deprotonation energy is now significantly higher than all previously calculated deprotonation energies on the different terminations of ZnO. Nevertheless, even in this extreme case, the deprotonation of MAX is still favorable, with a negative reaction energy. While the calculated energies for FAX are positive, they are still much lower than those for the gas phase. The deprotonation in both cases is achieved with a proton transferring from the organic perovskite precursor cation to an OH that resulted from the partial water dissociation on the fully water-covered ZnO surface.

In the case of MAX, we also observe that after this deprotonation step, a water molecule desorbs spontaneously and a halide atom is adsorbed on the Zn atom that is left exposed. Although this spontaneous desorption was not observed for FAX, we expect a qualitatively similar behavior. For the hydroxylated/water-covered surfaces, the dissociation energies (reactions A2 and B2) were not calculated. These reactions are expected to be less favorable than deprotonation, as was the case for FAX on all of the studied surfaces and for MAX on most of the studied surfaces. Still, even the fully water-covered (101̅0) ZnO surface seems to facilitate the degradation of the organic perovskite precursors, as the pristine nonpolar and polar surfaces and the nonpolar surface with O vacancies, although to a lesser extent and mainly through deprotonation.

## Discussion

Based on the computational results, we can now make a correlation with the experimental results presented in Figures 1 and 2. Our solution-processed ZnO surface is obviously not as well-defined as any of the model surfaces discussed above. In the prepared ZnO samples, we expect to have a combination of polar as well as nonpolar terminations, vacancies, and other defects, as well as surface contamination. Contaminants include the C–O and O–C=O carbon species mentioned in the discussion of Figure 2 as well as surface OH groups shown in Figure 1a. The adsorbed water studied in the calculations is not observed in our experiments. We suggest that any water molecules that might be present can desorb under ultrahigh vacuum (UHV) conditions in combination with X-ray exposure, whereas adsorbed H and OH may be more stable.

From the measurements shown in Figure 2, we know that none of the organic precursors remain intact when deposited on ZnO. Here, volatile species as well as new surface bonds are created, which are listed in Table 3. The nitrogen species N^1^, seen for all precursor-exposed samples, is likely related to NH_3_, created in the dissociation reactions (A2/B2), as it is the only common nitrogen-containing reaction product. A similar binding energy for NH_3_ on NiO has been reported before.^52^ The species N^2^, which is only present for FAX, must be related to the degradation product HCN. This association is supported not only by a good agreement of the observed binding energy with previous XPS reports for HCN,^53^ but also by the fact that the additional carbon peak C^2^, which is also only observed for FAX, has approximately the same intensity after correcting with the relative sensitivity factors. Both signals therefore belong to HCN; however, it is also possible that this species is adsorbed as CN^–^ after the deprotonation of this strong acid. The origin of the common C^1^ peak is less clear. For MAX this is likely the CH_3_ species absorbed on surface oxygen. This should, however, not be present for FAX, where a similar peak is observed. We speculate that for the FAX samples, the C^1^ belongs to physisorbed FA, created in the deprotonation reaction, which could exhibit a similar binding energy. As a consequence, also part of the N^1^ signal for the FAX samples must originate from physisorbed FA.

**Table 3:** Overview of Observed Physisorbed Species Indicated in Figure 2

peak	*E*_B_	physisorbed species
C^1^	∼288.8 eV	CH_3_, FA
C^2^	∼287.6 eV	HCN
N^1^	∼400.5 eV	NH_3_, FA
N^2^	∼399 eV	HCN

Therefore, we can conclude that some of the FA sticks to the surface in addition to the HCN/CN^–^, while no MA is seen for the MAX samples; for this latter species the N signal would have to appear at a significantly higher binding energy than N^1^. The volatility of MA is the reason why the experiments show that more N signal is lost for MAX than for FAX. Based on the adsorption energies in Table S1 in the Supporting Information, FA adsorbs more strongly, which supports the experimental observations.

For the halides our experiments also confirm the results found by DFT. We observe that I^–^ and Br^–^ strongly bind to the Zn atoms at the surface, leading to high halide signals in XPS. This is in contrast to our previous study on MoO_3_, where the I^–^ underwent a redox reaction with the Mo on the surface, leading to its oxidation and the formation of volatile I_2_.^29^

Finally, we have to come back to Figure 1d, in which we observed a lowering of the number of OH groups on the ZnO surface after treatment with the precursors. Based on Figure 4, rather an increase in OH groups would be expected since HI or HBr dissociates, forming new OH surface bonds. However, in the discussion of the hydroxylated surface above, it was found that the deprotonation reaction results in the adsorption of a proton on top of OH, forming H_2_O. The creation of volatile water is likely the reason for the observed reduction in OH surface density upon treatment of ZnO with the precursors due to this reaction pathway.

Both XPS and DFT, therefore, clearly revealed that the surface of ZnO is not suitable for direct contact with the organic halide perovskites. Its basic nature causes the decomposition of the organic precursors, which is only weakly affected by the surface termination, the presence of defects, or the presence of adsorbed water/OH. This indicates that independent of the preparation method, bare ZnO is unsuitable as the charge extraction layer here. Interestingly, Tsarev et al.^54^ reported that a surface treatment with MAI before the deposition of the perovskite led to a mitigation of the interfacial reactivity. Since we have seen in our study that MAI deprotonates and HI binds to the surface, we used our DFT model to also explore the effect of halogen coverage of ZnO on the reactivity toward the precursors; a discussion on such a passivated surface can be found in the Supporting Information, Figure S9. In agreement with the experimental findings by Tsarev et al.,^54^ our calculations show that the subsequent deprotonation of MA^+^ is indeed unlikely on both the HI as well as a HBr covered (101̅0) ZnO surface. An approach to reduce the interface reactivity could therefore be to halogenate the ZnO surface or use excess halide in the precursor mixture. It has also been reported by Schutt et al. that the use of a FA/Cs-based perovskite enhances the stability,^55^ likely due to a better stability of the inorganic cation Cs^+^ that has not been studied here.

## Conclusions

In this work, the interaction between the organic halide perovskite precursors (MAI, FAI, MABr, and FABr) and the surface of ZnO was investigated. Experimentally, we observed a complete degradation of all four precursors on this surface by XPS, evident from the change in the C 1s and N 1s binding energy, which indicates the formation of degradation products. Significant losses in N and C species show that also volatile species are created here, while for the halide, no loss or even an accumulation at the interface was found, indicative of the formation of new surface bonds. The oxidation state of Zn does not change due to the interaction with the precursors, so no redox reactions are taking place. Using DFT, we studied two different decomposition reactions (deprotonation and dissociation) for each of the organic precursors on the three most commonly observed surface terminations, the nonpolar (101̅0) as well as the polar Zn-terminated (0001̅) and O-terminated (0001) ZnO. Indeed, we find that the decomposition of the precursors is very likely, mainly through deprotonation, but the more complex dissociation reactions can also happen. The different ZnO terminations are equally reactive and exhibit similar trends, whereby the order of reactivity of the precursors is MAI ≈ MABr > FAI ≈ FABr.

In addition, we looked at two modified ZnO surfaces, as these are likely to occur under realistic experimental conditions. In one case, we introduced O vacancies, and in the other case, the surface was covered with water/OH. The presence of O vacancies has only a minor effect on the calculated reaction energies, while for the fully water/OH covered surface, deprotonation becomes less likely, though it remains much more likely than in the gas phase. Overall, it can be concluded that the basic nature of ZnO seems to be incompatible with the organic perovskite precursors, independent of the choice of the halide or organic cation. Since a change in surface termination or the presence of vacancies did not have a significant influence on the reaction energies, it is also unlikely that the reactivity of ZnO can be significantly changed by modifying the processing conditions. It is likely necessary to insert buffer layers, such as fullerenes, between the ZnO and the perovskite film to avoid direct contact between the two or to switch to inorganic Cs-containing perovskites.

## Experimental and Computational Details

Film preparation was done inside a nitrogen-filled glovebox. ZnO films were prepared by using a 0.5 M solution of zinc acetate dihydrate (Sigma-Aldrich) dissolved in ethanol. Into this solution, ethanolamine was added in a 1:1 molar ratio, and the solution was left to dissolve completely overnight. Next, the solution was spin-coated onto ITO-covered glass substrates at 2000 rpm for 60 s; afterward, the samples were annealed in air at 200 °C for 1 h, yielding a film of appropriately 20 nm thickness. To confirm the complete conversion of the precursor solution into ZnO, we performed combined thermal gravimetric analysis (TGA) and differential scanning calorimetry (DSC), which are included in the Supporting Information, Figure S10. For the precursor study, MAI, MABr, FAI, and FABr (Great Cell Solar Materials) were each dissolved in isopropanol at a concentration of 0.05 M. These were spin-coated onto ZnO substrates at 1500 rpm for 30 s and subsequently annealed in the glovebox at 80 °C for 1 h. For the unreacted precursors, the same solutions were deposited onto ITO substrates covered by either PEDOT/PSS (Clevios 4083, Heraeus) in the case of MAI and FAI or ZnCl_2_ (Merck) for MABr and FABr. These two different substrates had to be used since we were not able to find a common substrate that did not degrade any of the precursors. On the one hand, PEDOT/PSS was found to be highly reactive toward bromide and could therefore not be used with MABr and FABr. On the other hand, MAI and FAI degraded on the ZnCl_2_ sample; however, this is not because of the ZnCl_2_ itself, but rather because this material is to some degree soluble in the precursors’ solvent isopropanol. This way, the underlying ITO substrate was partially in contact with the MAI and FAI. This led to significant degradation for the iodide containing precursors, while this was not apparent for the bromide-containing ones.

XPS measurements were performed in an ultrahigh vacuum system (base pressure 1×10^–9^ mbar). All samples were transferred to the measurement system under nitrogen avoiding air exposure. A nonmonochromatic X-ray source (VG) with Al Kα excitation was used, having a photon energy of 1486.61 eV. The emitted photoelectrons were measured using a hemispherical analyzer (Specs Phoibos 100). A pass energy of *E*_p_ = 10 eV was used for the Br 3d, I 3d_5/2_, Zn 2p_3/2_, and O 1s peaks, while the weaker N 1s and C 1s signals were measured at *E*_p_ = 20 eV. The XPS data were fitted by Voight profiles using the software “XPSPEAK 4.1”.

DFT calculations were performed using the projector augmented wave method, as implemented in the Vienna ab-initio simulation package.^56−59^ The electronic exchange–correlation interaction was described by the functional of Perdew, Burke, and Ernzerhof within the generalized gradient approximation^60^ and energy and force convergence criteria of 10^–5^ eV and 0.02 eV/Å respectively were used in all calculations.

The nonpolar (101̅0) ZnO surface was modeled by a 10-layer slab, with an in-plane 4 × 2 supercell comprising 160 atoms, and a vacuum region of 20 Å separating the slabs. The polar surfaces were modeled by 8-layer slabs, with in-plane 4 × 4 supercells comprising 128 Zn and O atoms. The bonds at the bottom of the slabs were saturated using hydrogen pseudoatoms with a nuclear charge of +1/2 and +3/2 for the (0001̅) and the (0001) slab, respectively. A vacuum region of 15 Å separated the slabs. All calculations were performed using a 2 × 2 × 1 Γ-centered *k*-point grid and a kinetic energy cutoff of 500 eV. A dipole correction was employed to avoid interaction between periodic images.^61^

The adsorption energies of the perovskite precursors and their dissociation products on the ZnO surface were calculated as
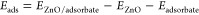
1where *E*_ZnO/adsorbate_, *E*_ZnO_, and *E*_adsorbate_ are the DFT total energies of ZnO with adsorbed species, the clean ZnO surface, and the adsorbate molecules, respectively.

## Supporting Information Available

Additional XPS data, DFT-based atomistic representations of the additional ZnO surfaces, table of calculated adsorption energies, calculation of surface passivation, and TGA/DSC measurements (PDF)
