# Supplementary material for: Probing the Reactivity of ZnO with Perovskite Precursors
Source: ACS Appl Mater Interfaces. 2024 Mar 14;16(12):14984–94. doi: 10.1021/acsami.4c01945 (PMC10983006; doi:10.1021/acsami.4c01945)
Supplement: Supplementary file 1 — am4c01945_si_001.pdf [file am4c01945_si_001.pdf]

Supporting Information

Probing the Reactivity of ZnO with Perovskite Precursors

Sofia Apergi,[1,2] Geert Brocks,[1,2,3] Shuxia Tao*,[1,2] Selina Olthof* [4]

[1] Materials Simulation and Modelling, Department of Applied Physics, Eindhoven University of Technology, P.O. Box 513, 5600 MB Eindhoven, The Netherlands
[2] Center for Computational Energy Research, Department of Applied Physics, Eindhoven University of Technology, P.O. Box 513, 5600 MB Eindhoven, The Netherlands
[3] Computational Chemical Physics, Faculty of Science and Technology and MESA+ Institute for Nanotechnology, University of Twente, P.O. Box 217, 7500 AE Enschede, The Netherlands
[4] University of Cologne, Institute for Physical Chemistry, Greinstrasse 4-6, 50939 Cologne, Germany

E-mail: S.X.Tao@Tue.nl, selina.olthof@uni-koeln.de

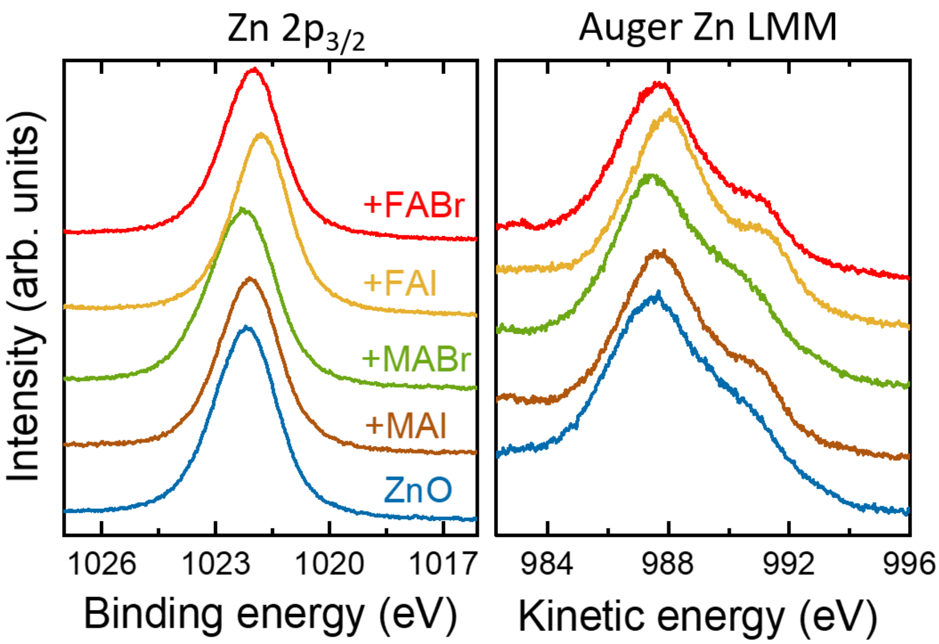

Figure S1: XPS measurements of the Zn $2p_{3/2}$ core level and Zn LMM Auger excitation for the as-prepared sample (same data as shown in Figure 1b), as well as ZnO exposed to the different precursors, as indicated in the Figure. The peak maxima extracted from these data sets were used in the Wagner plot in Figure 1c.

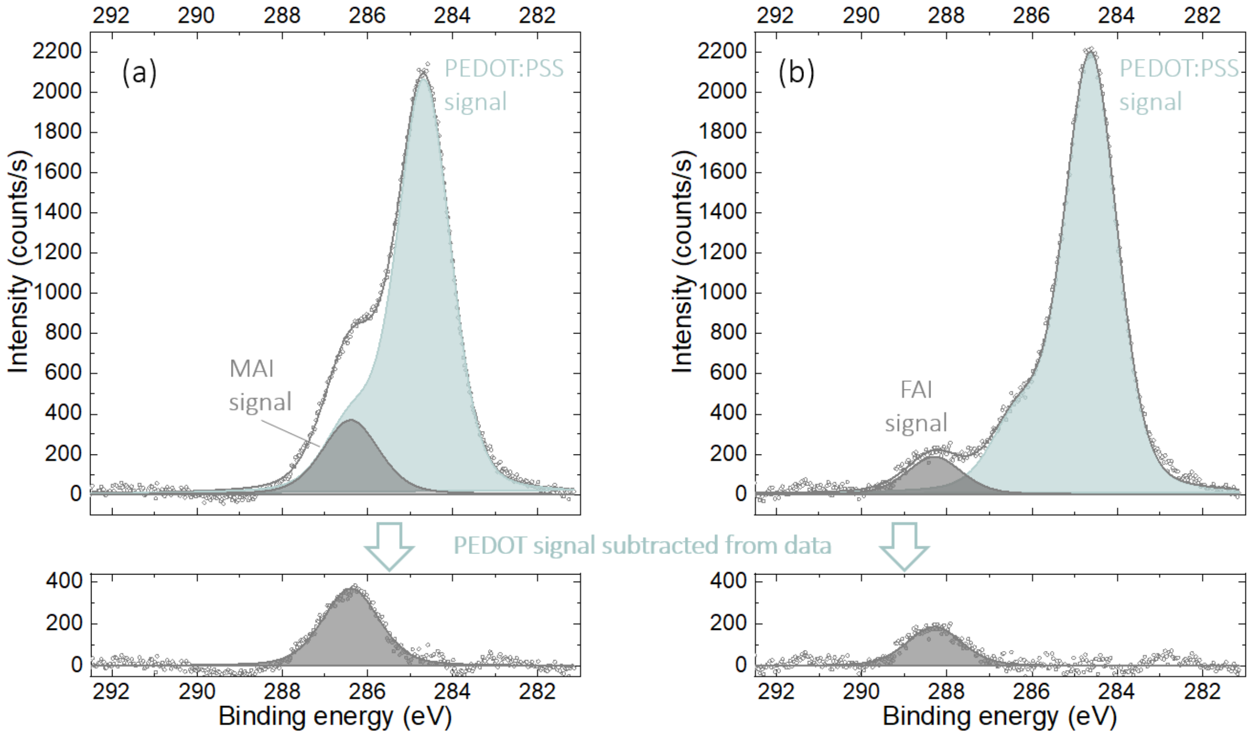

Figure S2: Carbon signal of the intact precursor measurements on PEDOT:PSS. a) The upper panel shows the as-measured data, the two shaded areas indicate the signal coming from PEDOT:PSS as well as MAI. The lower panel shows the same data once the PEDOT:PSS signal was subtracted, in order to better visualize the pure MAI signal. This modified data is included in Figure 2 in the main article. b) Same plot for the precursor FAI on PEDOT:PSS.

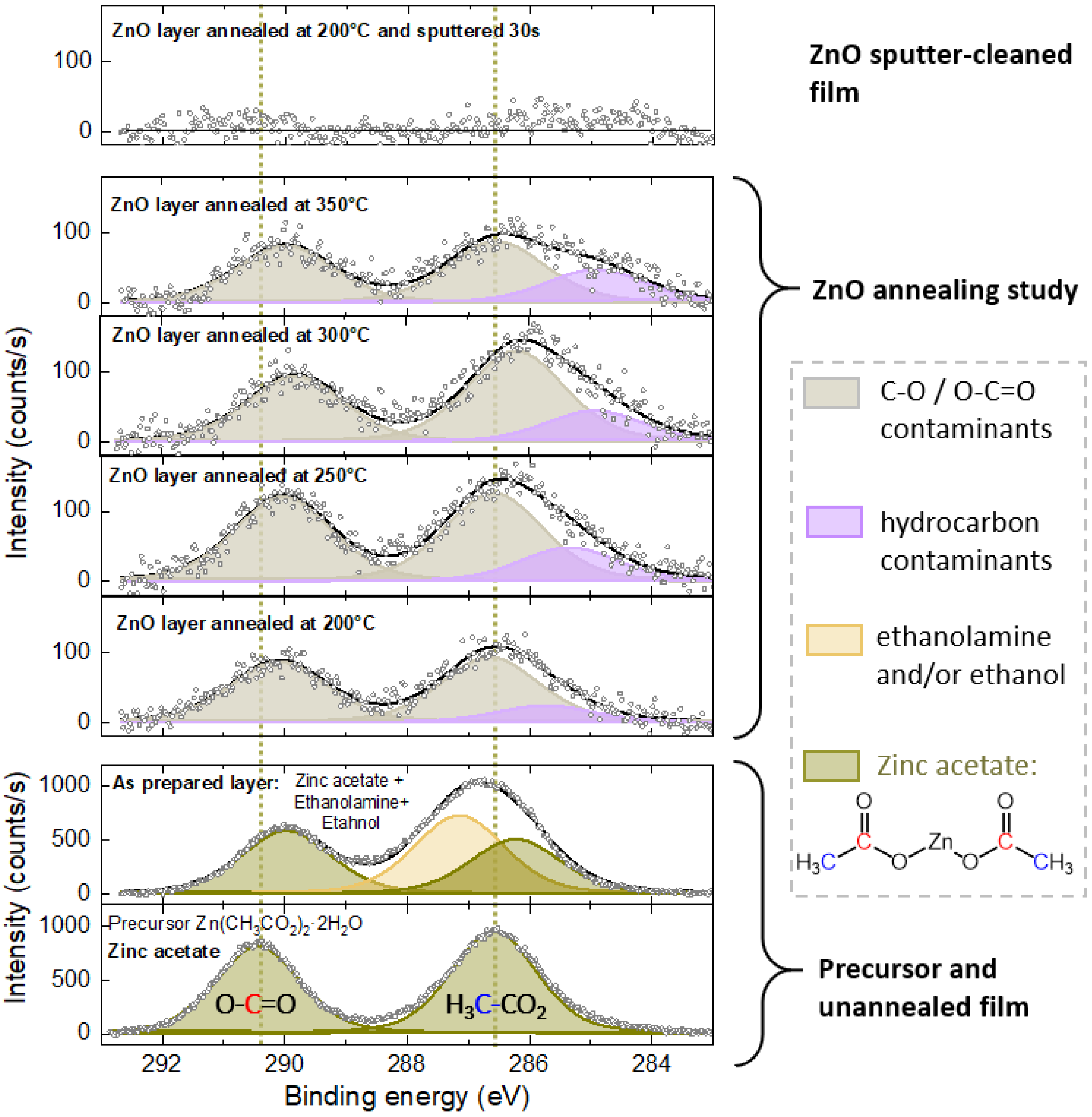

Figure S3: XPS data of the C1s region of various precursor and ZnO films. These measurements were performed in order to figure out the origin of the carbon signals on the ZnO surface. The lowest panel shows the C1s region of a thin film of zinc acetate dihydrate (after drying at 80°C in N_2). The two carbon signals of equal intensity can be associated with the two distinct carbon bonding environments in the zinc acetate molecule as indicated, see structure on the right. The second panel (from the bottom) shows a zinc acetate dihydrate : ethanolamine film without annealing, therefore the film is not converted into ZnO yet. The C1s bonds known from zinc acetate are still present, though slightly shifted, likely due to a difference in work function. In addition, the yellow marked feature appears that can be associated with ethanolamine and/or

remaining ethanol traces in the film. Upon annealing to 200°C (third panel) the amount of carbon significantly decreases (note the different y-axis scale), due to the formation of ZnO. Here, three carbon features remain. The one at the highest binding energy (~290 eV) is likely again a carboxyl bond (O-**C**=O), while the one at 286 eV is either the corresponding H_3**C**-CO_2 (same as in the acetate) or newly formed H_3**C**-O surface bond, which would appear at similar binding energy. The newly formed third peak is located around 285eV, a value typical for hydrocarbon contamination. It should be noted that these films have been annealed in air to convert to ZnO, so the adsorption of adventitious carbon is expected.

As bonds similar to zinc acetate remained on surface, possibly indicating an incomplete conversion of zinc acetate to ZnO, higher annealing temperatures of 250, 300, and 350°C were also explored and are included in this Figure. There is no significant effect on the carbon signals due to the annealing. This indicates that these carbon species do not originate from unreacted precursor, but rather form due to the air exposure during annealing. If this is the case, we only expect these species to be present on the surface, and not in the bulk of the film. To test this, a 200°C annealed ZnO sample was gently sputter cleaned in-situ for 30 s by an argon sputter gun (argon pressure $4\cdot10^{-6}$ mbar, sputter voltage 2 kV); approximately 5 - 10 nm of the surface is removed in this process. The C1s region after sputtering is included as the top panel and clearly shows the absence of carbon species in the bulk. This is a strong indication, that the carbon which is observed for the ZnO samples is exclusively located on the surface and indeed originates from contamination due to air exposure during annealing.

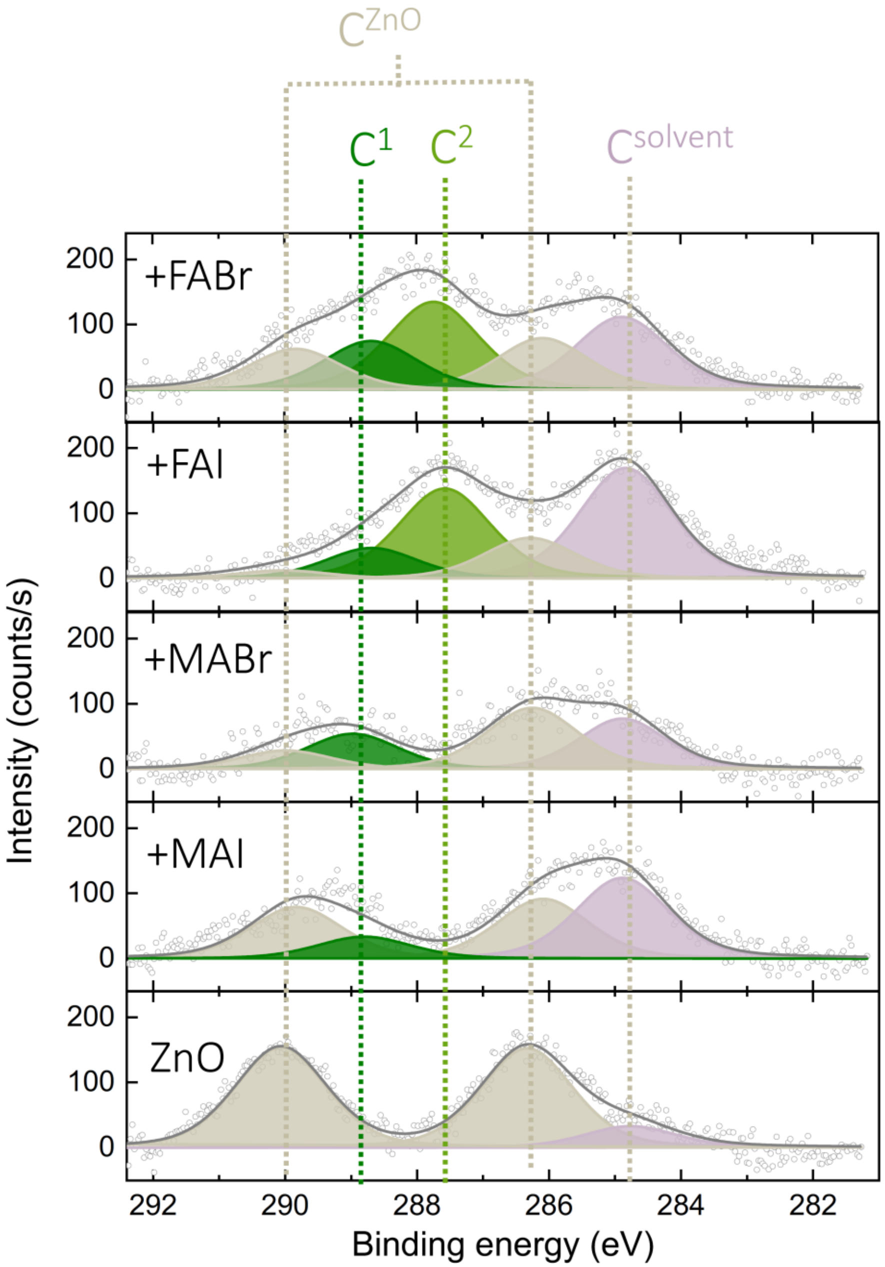

Figure S4: Larger and comparative view of the XPS data of the C 1s region. The upper 4 panels are the same plots as included in Figure 2 in the main article. The lowest panel is a pure ZnO annealed at 200°C, similar to the one included in Figure S3 above. The two carbon surface , which are discussed in more detail in the Figure S3, are labeled here as C^{ZnO}. The same peaks are still visible in the precursor exposed samples, since the overlayers are thin enough to still observe the substrate peaks.

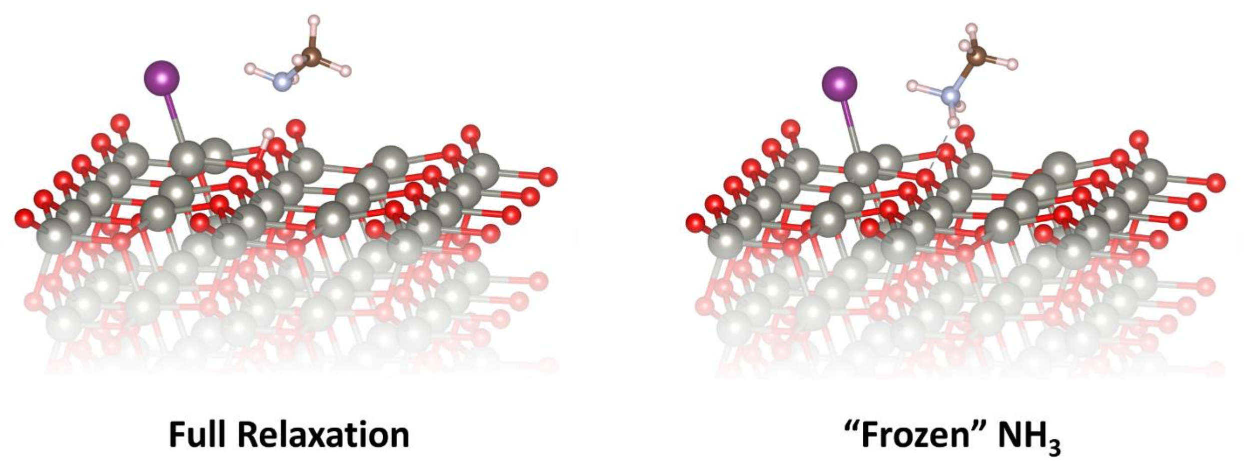

Figure S4: On the pristine polar ($000\bar{1}$) ZnO surface, MA^+ deprotonates spontaneously, when the structure is relaxed fully. In order to calculate the deprotonation energy we "freeze" the NH_3 part of MA+ and relax the positions of all other atoms.

Table S1: Adsorption Energies in eV of the Precursor Molecules and of the Decomposition Products on the Clean ZnO ($10\bar{1}0$), ($000\bar{1}$), and (0001) Surfaces.

	Adsorption Energy (eV)			
	($10\bar{1}0$)	($000\bar{1}$)	(0001)	($10\bar{1}0$) O Vac.
**Precursor Molecules**				
MAI	−1.21	−1.47	−2.85	−1.21
FAI	−1.53	−1.69	−2.26	−1.53
MABr	−1.21	−1.45	−2.63	−1.21
FABr	−1.50	−1.66	−2.07	−1.50
**Reaction Products**				
Methylamine	−1.25	−0.91	−1.62	−1.25
Formamidine	−1.56	−1.01	−2.20	−1.56
HI	−2.09	−2.92	−2.78	−2.09
HBr	−1.97	−2.82	−2.16	−1.97
NH_3	−1.19	−0.85	−1.09	−1.19
CH_3I	−0.95	−3.07	−2.01	−0.95
CH_3Br	−0.93	−3.08	−1.53	−0.93
NH_4I	−2.84	−3.28	−3.31	−2.84
NH_4Br	−2.67	−3.14	−2.65	−2.67
HCN	−0.59	−0.34	−0.12	−0.59

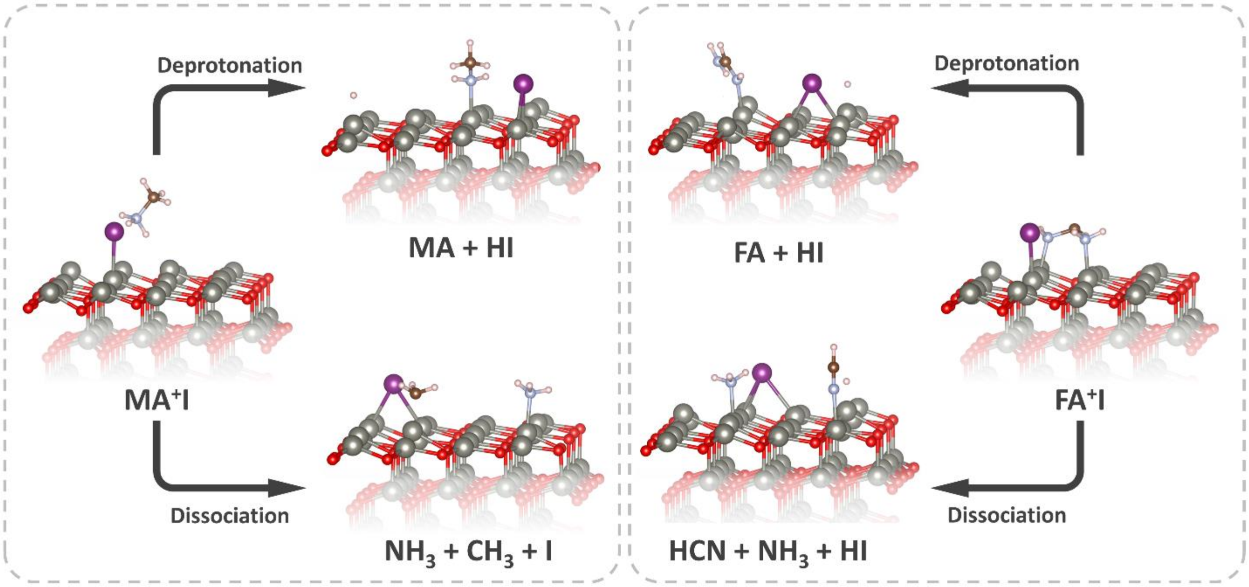

Figure S6: Atomistic representation of reactions on the surface of pristine polar $(000\overline{1})$ ZnO.

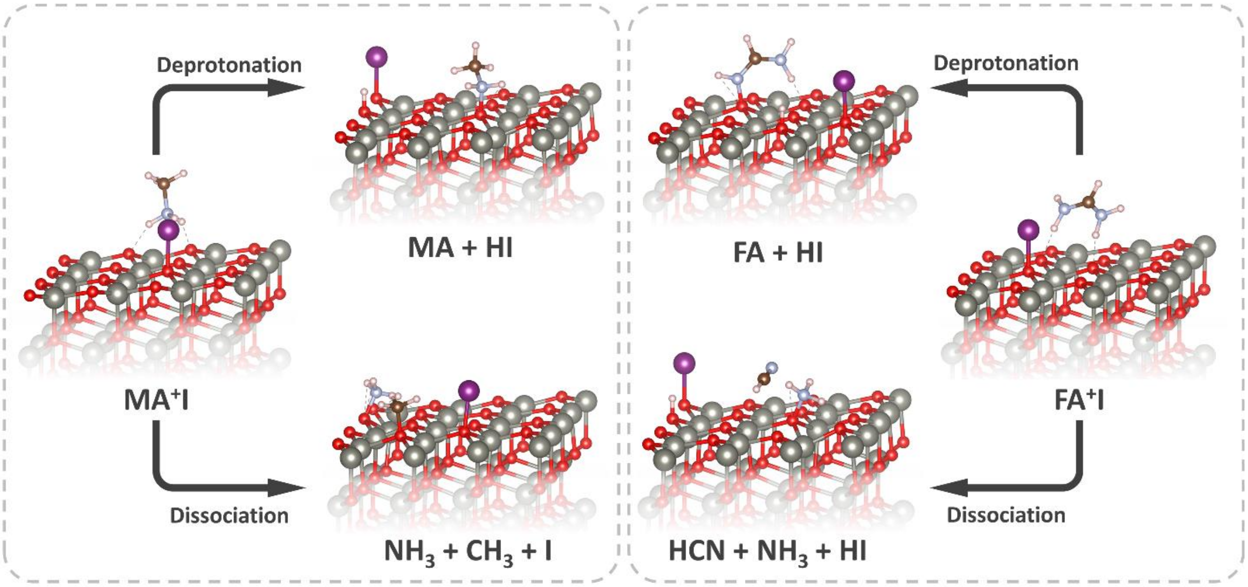

Figure S7: Atomistic representation of reactions on the surface of pristine polar (0001) ZnO.

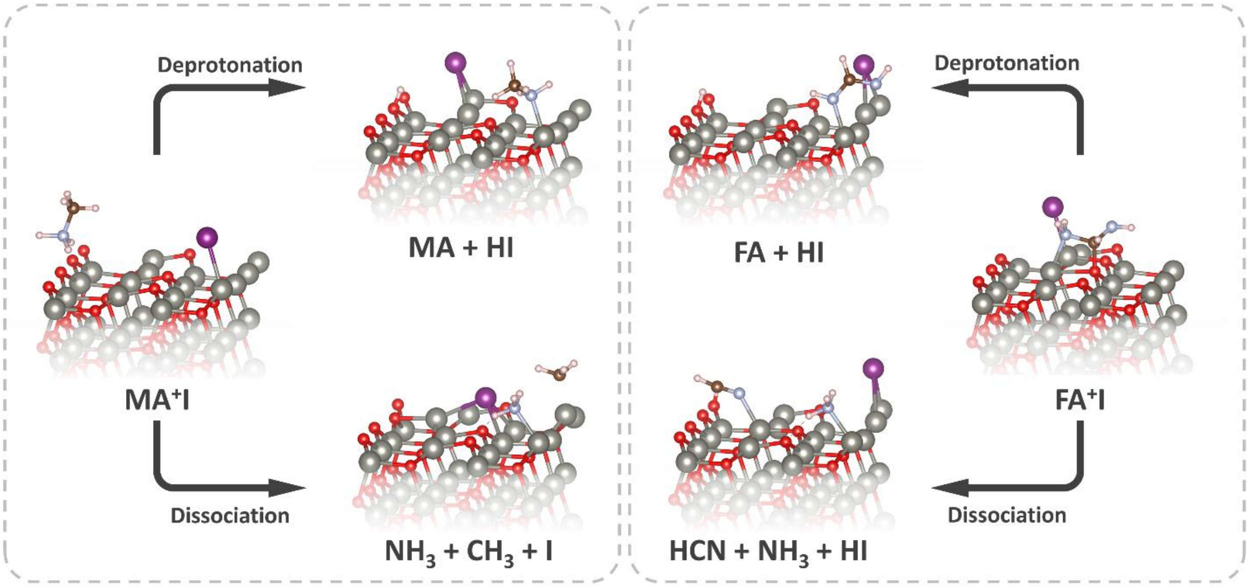

Figure S8: Atomistic representation of reactions on the surface of non-polar ($10\bar{1}0$) ZnO surface with an O vacancy.

Surface Passivation

To investigate the ability of MAX to passivate the surface of ZnO, we calculate the deprotonation energies of the organic precursors on a HX covered $(10\bar{1}0)$ ZnO surface. To calculate the deprotonation energy for each molecule we use two structures; in the initial structure MAX are put on top of the HX covered ZnO surface, while in the final structure we move one proton from the organic cation on the surface of the oxide. We then relax the structures and calculate their energy difference. However, as can be seen in Figure S8, when a neutral MA or FA molecule is put on top of a HI covered surface and the structure is relaxed, the result is the organic molecule attracting one of the surface protons. The same happens on a HBr covered surface. This indicates that the deprotonation of MA^+ is actually unlikely in the case of a halide covered surface.

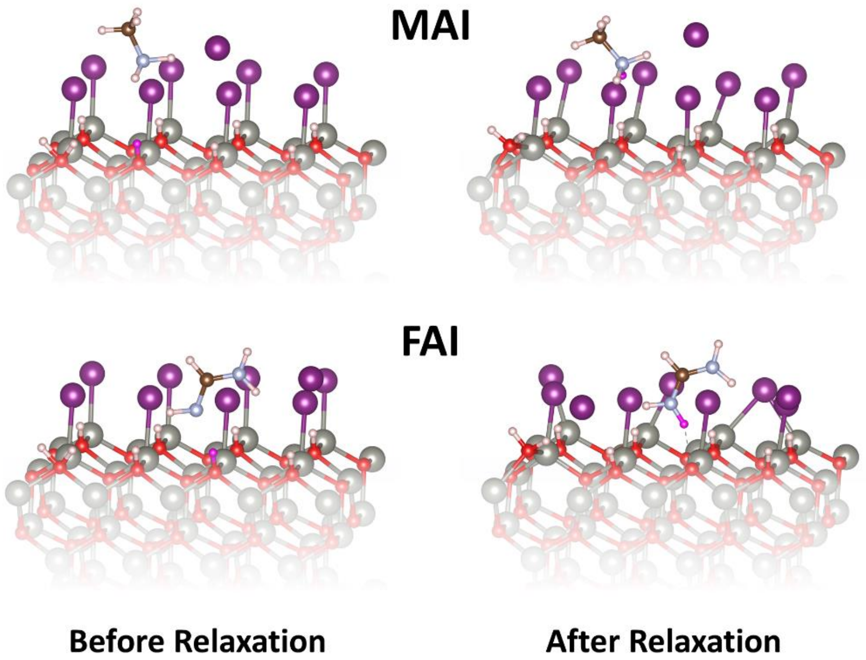

Figure S9: Atomistic representation of the interaction between the HI passivated $(10\bar{1}0)$ surface and the neutral molecules MA. After the structures are relaxed, the neutral molecules have adsorbed one of the surface H atoms.

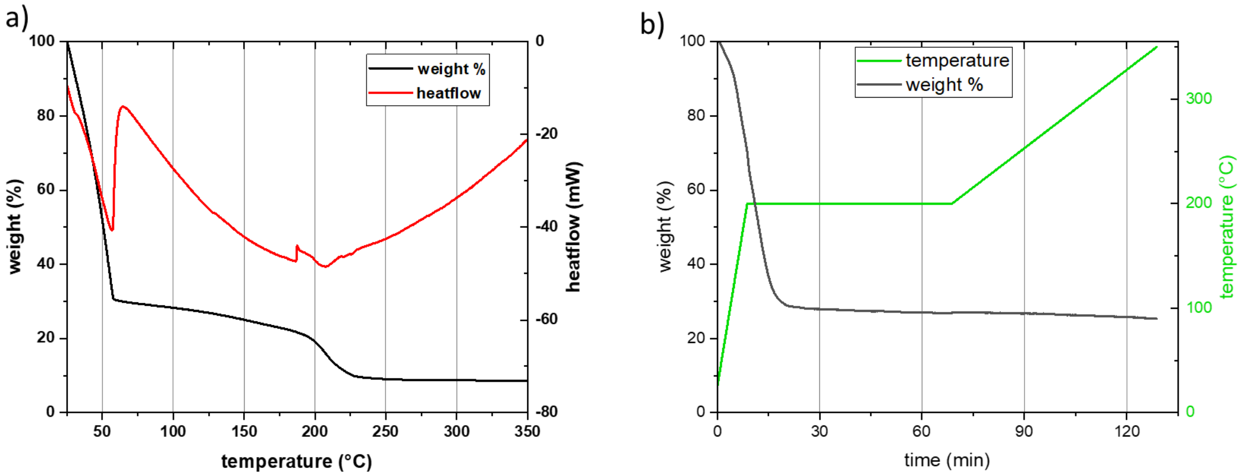

Figure S10: TGA and DSC analysis of the prepared ZnO films. a) Combined TGA and DSC analysis of 50 µL of the zinc acetate dihydrate:ethanolamine precursor solution using a temperature ramp 3K/min. The initial weight loss up to 60°C is due to ethanol evaporation. From approximately 190°C on, an additional weight loss is seen, due to the formation of ZnO. b) TGA analysis of an annealing similar to the thin-film ZnO preparation in the main article: Here, the temperature (green line) of the precursor solution was ramped quickly (within 8 min) to 200°C and then held at this value for the standard annealing time of 1h. From the weight loss, it can be seen that the ongoing reaction is completed within approximately 20 min. The subsequent heating step to 350°C is not related to the sample preparation in the main article. This was done to look out for any additional weight losses that could indicate an incomplete ZnO formation at 200°C. As no additional weight loss is observed, we can conclude that the conversion of the zinc acetate to ZnO has been completed during the previous 200°C annealing step
